# Towards automated extraction of 2D standard fetal head planes from 3D ultrasound acquisitions: A clinical evaluation and quality assessment comparison

**DOI:** 10.1016/j.radi.2020.11.006

**Published:** 2021-05

**Authors:** E. Skelton, J. Matthew, Y. Li, B. Khanal, J.J. Cerrolaza Martinez, N. Toussaint, C. Gupta, C. Knight, B. Kainz, J.V. Hajnal, M. Rutherford

**Affiliations:** a Perinatal Imaging Department, King's College London, UK; b School of Biomedical Engineering and Imaging Sciences, King's College London, UK; c Department of Computing, Imperial College London, UK; d Guy's & St Thomas' NHS Foundation Trust, UK

**Keywords:** Clinical evaluation, Fetal imaging, Quality assessment, Ultrasound

## Abstract

**Introduction:**

Clinical evaluation of deep learning (DL) tools is essential to compliment technical accuracy metrics. This study assessed the image quality of standard fetal head planes automatically-extracted from three-dimensional (3D) ultrasound fetal head volumes using a customised DL-algorithm.

**Methods:**

Two observers retrospectively reviewed standard fetal head planes against pre-defined image quality criteria. Forty-eight images (29 transventricular, 19 transcerebellar) were selected from 91 transabdominal fetal scans (mean gestational age = 26 completed weeks, range = 20^+5^–32^+3^ weeks). Each had two-dimensional (2D) manually-acquired (2D-MA), 3D operator-selected (3D-OS) and 3D-DL automatically-acquired (3D-DL) images. The proportion of adequate images from each plane and modality, and the number of inadequate images per plane was compared for each method. Inter and intra-observer agreement of overall image quality was calculated.

**Results:**

Sixty-seven percent of 3D-OS and 3D-DL transventricular planes were adequate quality. Forty-five percent of 3D-OS and 55% of 3D-DL transcerebellar planes were adequate.

Seventy-one percent of 3D-OS and 86% of 3D-DL transventricular planes failed with poor visualisation of intra-cranial structures. Eighty-six percent of 3D-OS and 80% of 3D-DL transcerebellar planes failed due to inadequate visualisation of cerebellar hemispheres. Image quality was significantly different between 2D and 3D, however, no significant difference between 3D-modalities was demonstrated (p < 0.005). Inter-observer agreement of transventricular plane adequacy was moderate for both 3D-modalities, and weak for transcerebellar planes.

**Conclusion:**

The 3D-DL algorithm can automatically extract standard fetal head planes from 3D-head volumes of comparable quality to operator-selected planes. Image quality in 3D is inferior to corresponding 2D planes, likely due to limitations with 3D-technology and acquisition technique.

**Implications for practice:**

Automated image extraction of standard planes from US-volumes could facilitate use of 3DUS in clinical practice, however image quality is dependent on the volume acquisition technique.

## Introduction and literature review

The fetal head and brain is examined during 18^+0^-20^+6^ fetal anomaly ultrasound (US) examinations to assess growth and development of the skull and intracranial structures.^1^ Transventricular (TV) and transcerebellar (TC) views are routinely assessed in the basic screening examination. These two-dimensional (2D) planes allow identification of intracranial landmarks and acquisition of specific biometric measurements which, if absent or outside expected reference ranges, may be indicative of an anomaly.^2^ Occasionally, it is not possible to obtain these planes, resulting in an incomplete or suboptimal assessment.^3^

Advances in three-dimensional US (3D-US) and multi-planar reconstruction can complement conventional 2D-US and overcome some of its limitations.^4^ The 3D-transabdominal “single-shot” technique can be used to acquire a reproducible fetal head US-volume,^5^^,^^6^ with reconstructed images enabling detailed retrospective review by clinicians when multiple datasets are acquired from different insonation angles (e.g. transverse, sagittal, coronal).^7^ Biometric measurements from 3D-images have also demonstrated good correlation with 2D-methods.^3^

Visualisation of intracranial structures using 3D-US can be superior to 2D-US as reviewers are able to manipulate the image planes for optimisation,^6^ although this process can be time-consuming, requiring additional training in 3D-techniques and experience with different manufacturer's platforms, therefore is not feasible in clinics where real-time assessment is required.^8^ A deep learning (DL) algorithm to automatically-extract required standard planes from 3D-volumes^9^ could overcome these barriers, and facilitate the accessible use of 3D-techniques within US screening clinics.

Robust clinical evaluation of DL tools is essential to build on reported technical accuracy metrics as part of the clinical translation process.^10^ However, for fetal US, quality assessment (QA) is laborious and subjective because of variation in screening programmes and a lack of agreement on quantitative assessment criteria.^11^ Building on the work of Li et al.,^9^ this study aims to clinically evaluate the quality of automatically extracted standard TV and TC fetal head planes from 3D-US volumes in comparison to standard planes that are manually-acquired from 2D-US and operator-selected from 3D-US.

## Methods

Data was acquired between 2016 and 2019 as part of the Intelligent Fetal Imaging and Diagnosis (iFIND) project (NRES reference number = 14/LO/1086 and 07/H0707/105)*.* Participants gave informed written consent.

Inclusion criteria were: completed 18^+6^-20^+6^ clinical fetal anomaly scan and consent to fetal research imaging. Scans were undertaken by four operators (3 research sonographers, 1 obstetrician) (TF/JM/CK/ES) using a Philips EpiQ (Philips Healthcare, Best, Netherlands) US system with an X6-1 MHz matrix transducer to acquire the following image planes;

1. 2D-TV^1^
2. 2D-TC^1^
3. 3D-TV
4. 3D-TC.

3D-volumes were acquired from standard head planes (angle of insonation at 90° to mid-line echoes), using an acquisition sweep angle of 90° to ensure complete coverage of the fetal cranium.

### Algorithm development

**Figure 1:**
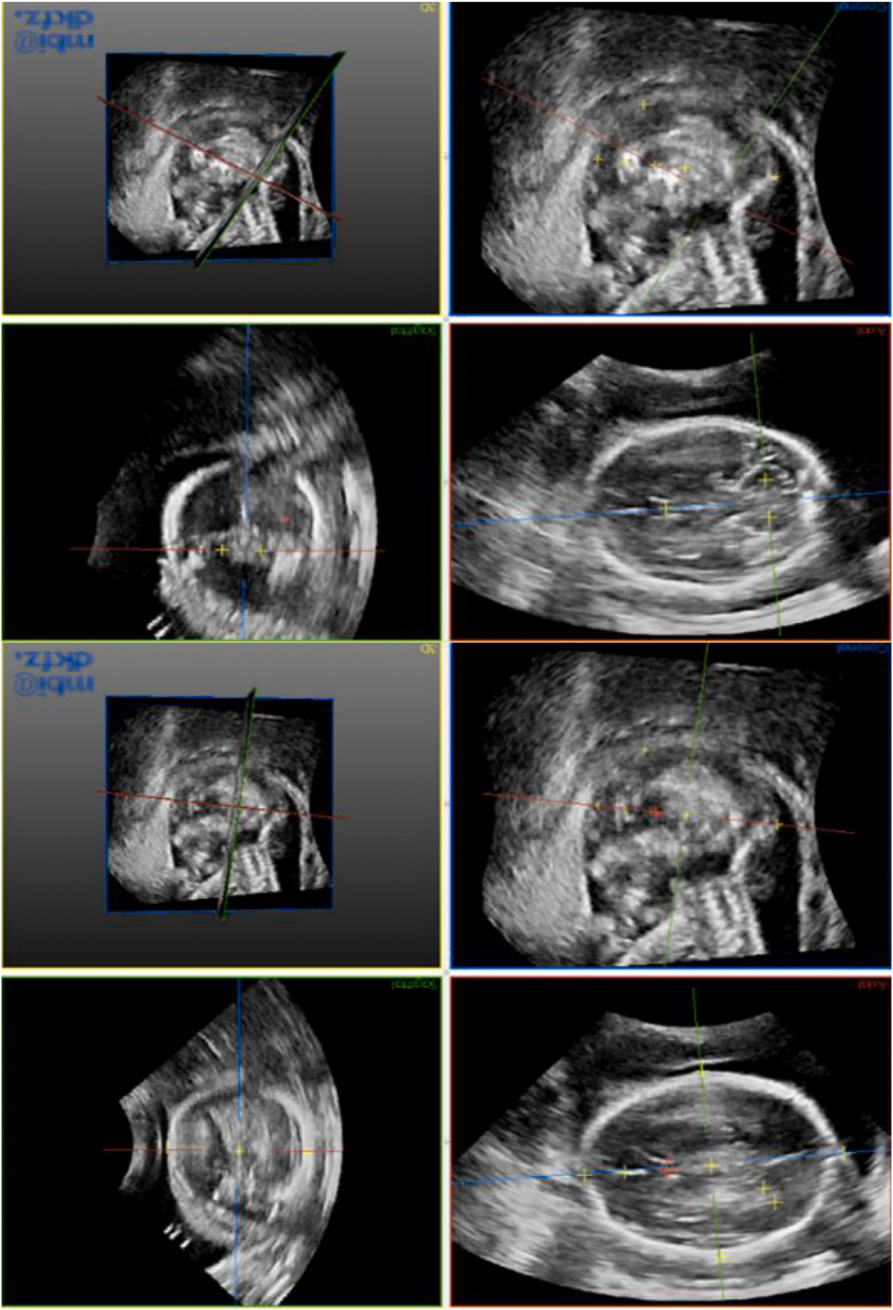
Landmark picking during manual annotation process in MITK workbench 2016.11. Anatomical landmarks as follows: 1- cavum septum pellucidum, 2- inner border of posterior horn of lateral ventricle, 3- outer border of posterior horn of lateral ventricle, 4- centre of bi-parietal diameter, 5- outer skull surface in near field, 6- outer skull surface in far-field, 7- occipital point of skull, 8- sinciput of skull, 9- mid-point between sinciput and cavum septum pellucidum, 10- mid-point of cerebellar hemisphere in near field, 11- mid-point of cerebellar hemisphere in far-field, 12- centre of left orbit, 13- centre of right orbit, 14- vertex of skull (image rotated to demonstrate familiar orientation of axial fetal head planes).

Using open-source software (MITK workbench 2016.11), 303 TV and 248 TC-planes were manually annotated by 3 observers (2 research sonographers, 1 medical student) (ES/JM/CG) to provide training data for tool development. For consistency, observers received in-person training by JM prior to annotating. The annotation process required the observers to identify 14 cranial landmarks (Fig. 1).

The tool is implemented using the M4+ Iterative Transformation Network (ITN) approach.^9^ ITN utilises a convolutional neural network to learn the mapping between a 2D-image and the rigid transformation required to move that plane towards the location and orientation of the standard plane in the 3D-volume.

**Figure 2:**
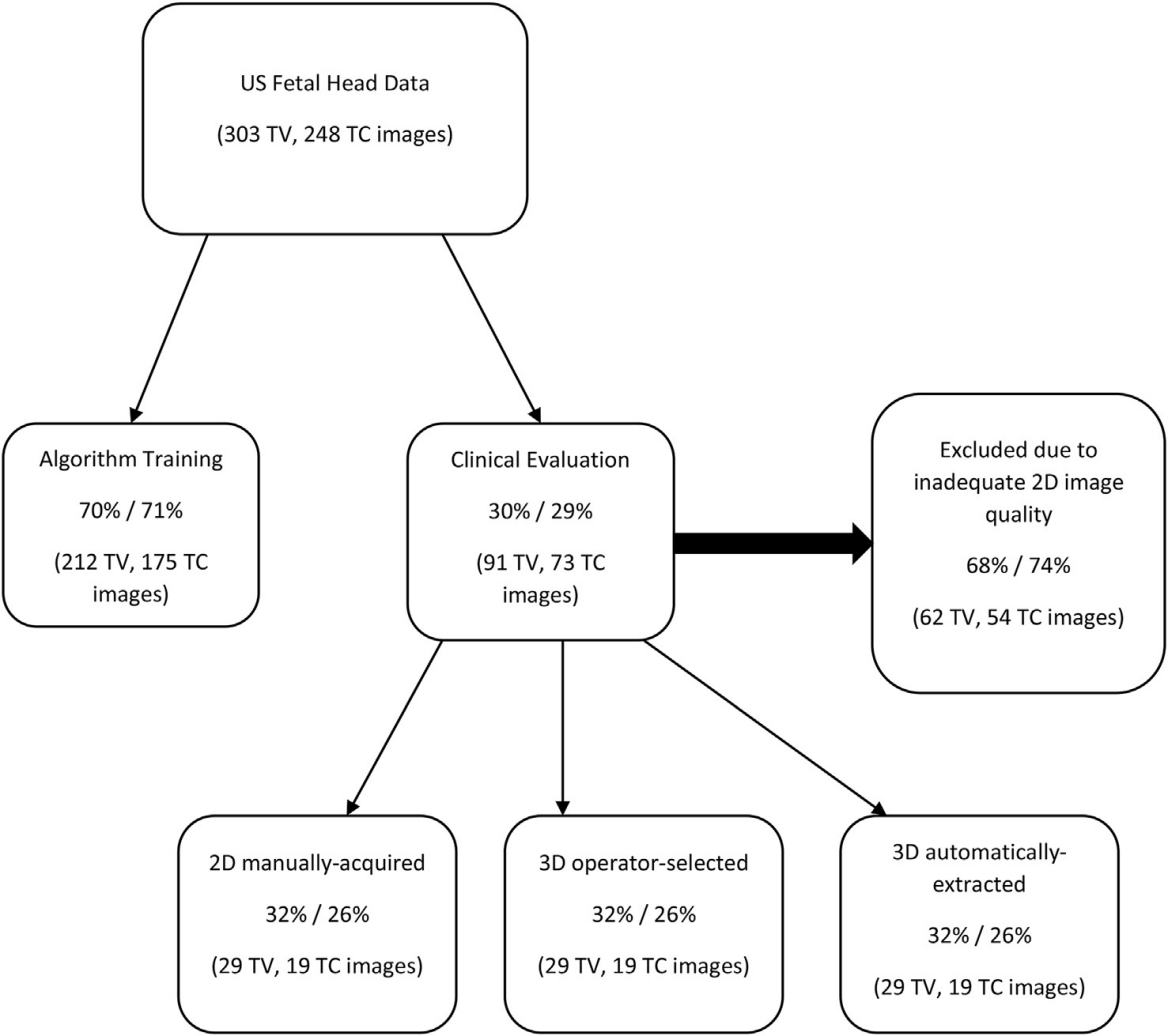
Study flowchart illustrating allocation of image data.

The dataset of manually annotated 3D-fetal head volumes was split into training and test sets (Fig. 2). There are fewer TC-plane annotations because of lack of available image data. Two separate models were trained for TV and TC-planes respectively.

The ITN model was implemented in Tensorflow^12^ running on a machine with Intel Xeon CPU E5-1630 at 3.70 GHz and one NVIDIA Titan Xp 12 GB GPU.^9^ Accuracy with the ITN model was evaluated quantitatively using: 1) distance between the plane centres (δx) and 2) rotation angle between the planes (δθ). On the test dataset, the ITN approach achieved a detection accuracy of δx=(3.68 ± 1.69)mm, δθ=(12.5 ± 6.1)° for TV-planes and δx=(3.69 ± 1.75)mm, δθ=(12.9 ± 6.9)° for TC-planes. These results show the ITN model can accurately predict both the location and orientation of the planes.

Using a randomisation tool developed in MATLAB (The MathWorks, USA), a selection of the remaining planes were presented for retrospective quality assessment (Fig. 2).

### Quality assessment

For quality assessment (QA), a software tool (USQA) and a quality scoring system was developed, devised from peer-reviewed studies and guidance by the NHS Fetal Anomaly Screening Programme.^1^^,^^11^^,^^13^, ^14^, ^15^, ^16^, ^17^ Two observers (>20-years combined ultrasound experience) (ES/JM) performing QA received prior formal USQA training from a software engineer (NT) using unrelated images. To minimise recall bias for the observers, a break of 4-weeks was included between annotation and QA. The observers were blinded to the plane's modality and instructed to perform a binary assessment of pre-defined criteria (Table 1) derived from existing literature^1^^,^^11^^,^^13^, ^14^, ^15^, ^16^ for each image, including a final assessment of overall adequacy. Not all criteria required a “pass” for the image to be considered adequate overall: some technical factors (e.g. hemisphere asymmetry) may not be sufficiently detrimental to render the image inadequate, but criterion related to visualisation of key anatomical landmarks for the standard plane (e.g. cavum septum pellucidum, CSP) are essential. For intra-observer agreement, both observers were presented with a sub-set (n = 25) of randomly selected images for re-review after 2-weeks.

**Table 1:** Pre-defined quality assessment criteria for TV and TC standard planes derived from existing literature.^1^^,^^9^, ^10^, ^11^, ^12^, ^13^

TV plane	TC plane
Symmetrical appearance of right-left hemispheres	Symmetrical appearance of right-left hemispheres
CSP well visualised	CSP well visualised
Posterior ventricle/choroid plexus well visualised	Equal size cerebellar hemispheres (borders not obscured by posterior fossa)
No cerebellum visualised	Occipital bone and nuchal skin fold visible
Oval skull shape > round skull shape	Well visualised intra-cranial structures
Well visualised intra-cranial structures	**Overall adequate view**
**Overall adequate view**	

Primary outcome measures were:

1. Overall adequacy of image quality from TV and TC-planes between three modalities: 2D manually-acquired (2D-MA), 3D operator-selected (3D-OS) and 3D-DL automatically-extracted (3D-DL)
2. Assessment of specific image features contributing to inadequate images
3. Assessment of intra ad inter-observer agreement of quality scoring.

### Statistical analysis

Data was analysed using SPSS (version 24, SPSS Inc, USA). Non-parametric statistical analysis was undertaken because of the categorical nature of the dataset. McNemar's test was used to determine any differences between the number of adequate planes per modality for each observer. Where there was inadequate image quality for 3D-planes, a sub-analysis was performed to identify the criteria contributing to lower quality. Inter-observer agreement strength was assessed using Cohen's Kappa, and the percentage intra-observer agreement was calculated (p < 0.05 was used to determine statistical significance).

## Results

### Participant demographics

Images from 91-TV and 73-TC cases were available for review. There were 29-TV and 19-TC 2D-MA images that observers agreed were of overall adequate quality. These were selected as the gold-standard for comparative analysis against their corresponding image from 3D-OS and 3D-DL. Mean maternal age at consent was 33 years (range 24–40). Mean maternal BMI was 27 kg/m^2^ (range 20.05–45.6 kg/m^2^). Mean gestational age (GA) at the time of scan was 26 completed weeks (range 20^+5^-32^+3^ weeks). There were 3 cases of fetal cardiac anomaly: 2 right aortic arch and 1 suspected coarctation of the aorta. Two additional cases had placenta praevia. No cases had any identifiable structural head or brain anomalies.

### Quality assessment of TV-planes (Table 2)

Of 29 TV-planes, observer-1 rated 62% of 3D-OS TV-planes as adequate. Observer-2 rated 72% of 3D-OS TV-planes as adequate. Of the planes that observer-1 rated inadequate (n = 11), observer-2 gave the same rating in 7 (64%).

**Table 2:** Observer assessment of overall TV-plane quality.

	2D manually-acquired and 3D operator-selected both adequate	2D manually-acquired and 3D-operator selected both inadequate	Total
Observer 1	18	11	29
Observer 2	21	8	29
Total	39	19	58
	2D manually-acquired and 3D-DL automatically extracted both adequate	2D manually-acquired and 3D-DL automatically-extracted both inadequate	
Observer 1	20	9	29
Observer 2	19	10	29
Total	39	19	58

For 3D-DL TV-planes, observer-1 rated 69% as adequate, and observer-2 rated 66% of as adequate. Of the planes that observer-1 rated inadequate (n = 9), observer-2 gave the same rating in 7 (78%). There were 3 cases (10%) where observers agreed that both the corresponding 3D-OS and 3D-DL TV-planes were inadequate.

### Quality assessment of TC-planes (Table 3)

Of 19 TC-planes, observer-1 rated 58% of corresponding 3D-OS planes as adequate. Observer-2 rated 32% as adequate. Of the planes that observer-1 rated inadequate (n = 8), observer-2 gave the same rating in 7 (88%).

**Table 3:** Observer assessment of overall TC-plane quality.

	2D manually-acquired and 3D operator-selected both adequate	2D manually-acquired and 3D-operator selected both inadequate	Total
Observer 1	11	8	19
Observer 2	6	13	19
Total	17	21	38
	2D manually-acquired and 3D-DL automatically extracted both adequate	2D manually-acquired and 3D-DL automatically-extracted both inadequate	
Observer 1	7	12	19
Observer 2	8	11	19
Total	15	23	38

For 3D-DL TC-planes, observer-1 rated 37% as adequate. Observer-2 rated 42% as adequate. Of the 3D-DL TC planes that observer-1 rated inadequate (n = 12), observer-2 gave the same rating in 9 (75%).

There were 5 cases (26%) where observers agreed that both the 3D-OS and 3D-DL TC-planes were inadequate.

### Sub-analysis of inadequate 3D planes

For inadequate 3D-OS TV-planes (n = 7), the image quality criterion with the highest proportion of agreed failures was visualisation of the CSP (71%). For 3D-OS TC-planes (n = 7), this was poor visualisation of the cerebellar hemispheres (86%).

**Figure 3:**
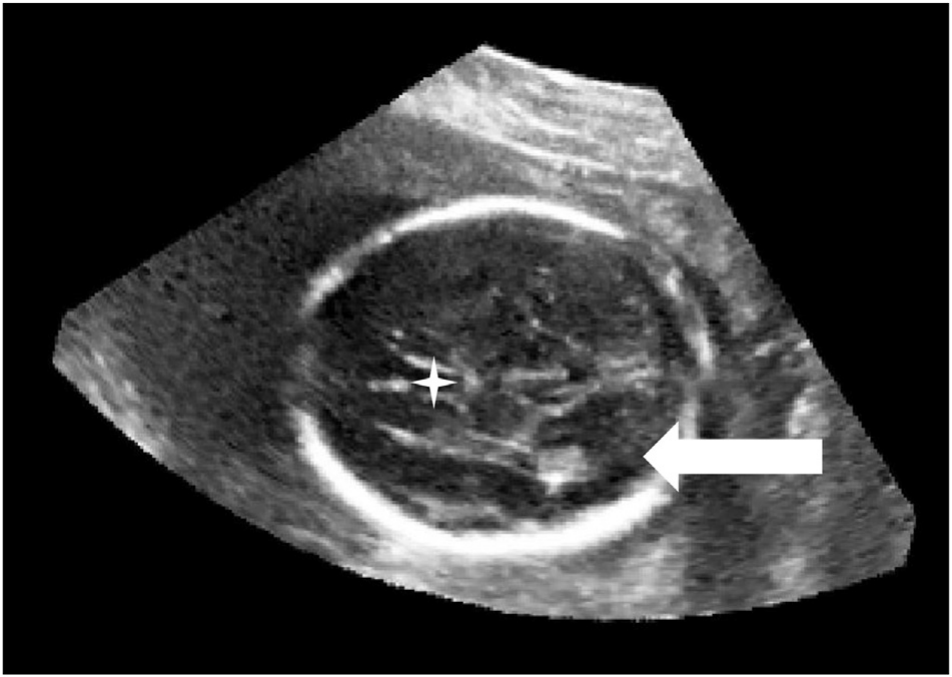
3D-DL automatically-extracted TV-plane rated as overall inadequate image quality due to poor visualisation of the posterior horns of the lateral ventricle (solid white arrow to demonstrate poorly visualised posterior horn, star indicates cavum septum pellucidum).

**Figure 4:**
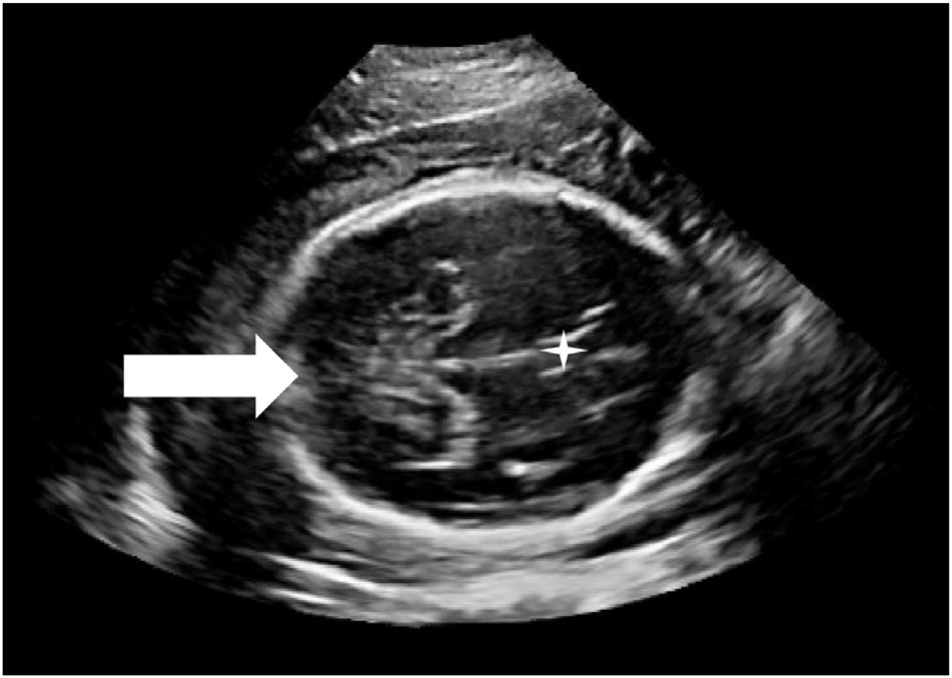
3D-DL automatically-extracted TC-plane rated as overall inadequate image quality due to poor visualisation of the cerebellar hemispheres (solid white arrow to demonstrate non-visualisation of cerebellar hemispheres in posterior fossa, star indicates cavum septum pellucidum).

For 3D-DL TV-planes (n = 7), the highest proportion of agreed failures was visualisation of the posterior horns of the lateral ventricle (86%) (Fig. 3). For failed 3D-DL TC-planes (n = 9), this was poor visualisation of the cerebellar hemispheres (80%) (Fig. 4).

### Observer agreement of image adequacy

Observers agreed 59% of 3D-OS and 3D-DL TV-planes were of overall adequate quality. Of these, there were 10 cases where both 3D-TV-planes were of overall adequate quality. Observers agreed 24% of 3D-OS and 10% of 3D-DL TC-planes included were of overall adequate quality. There was only one case where both 3D-TC-planes were overall adequate quality.

Cohen's kappa was calculated to assess the strength of the inter-observer agreement in the overall adequacy of the image achieved beyond chance as per McHugh (Table 4).^18^

**Table 4:** Cohen's kappa for inter-observer agreement of quality scoring per standard plane and modality.

Overall adequate view	Inter-observer agreement (Observer 1 and Observer 2)	
	3D operator-selected	3D-DL automatically-extracted
TV plane	Moderate (k = 0.613, p = 0.001)	Moderate (k = 0.609, p = 0.001)
TC plane	Weak (k = 0.486, p = 0.026)	None (k = 0.184, p = 0.419)

Inter-observer agreement of the overall adequacy of TV-planes was moderate in 3D-OS (k = 0.613) and 3D-DL (k = 0.609). Inter-observer agreement for the overall adequacy of TC-planes was weak for 3D-OS (k = 0.486). No inter-observer agreement in the overall adequacy of 3D-DL TC-planes was demonstrated. The power of these findings is uncertain because of the low number of cases.

Due to small intra-observer case numbers, it was not possible to statistically analyse the strength of intra-observer agreement, therefore, the percentage agreement was calculated.

Eleven TV-planes were included for intra-observer re-review: 2D-MA (n = 5), 3D-OS (n = 2) and 3D-DL (n = 4). Observer-1 had the highest agreement with 8 (72%) of TV-planes rated the same in both review periods. Observer-2 rated 6 (54%) of the included TV-planes as the same in both reviews.

The TC-planes included for intra-observer re-review were: 2D-MA (n = 1), 3D-OS (n = 3) and 3D-DL (n = 1). There were no agreed images for either observer.

### Comparison of imaging modalities per observer

McNemar's tests found significant differences in the overall adequacy of the 2D-MA planes compared to 3D as rated by both observers, however no significant difference between 3D-images was demonstrated (p < 0.005) (Table 5).

**Table 5:** Comparison of imaging modalities and overall adequacy of image per observer (McNemar's test). p < 0.05 was used to determine statistical significance.

Standard plane		3D operator-selected			
**TV**	**2D manually-acquired**	**Observer 1**		**Observer 2**	
		**Fail**	**Pass**	**Fail**	**Pass**
	**Pass**	11	18	8	21
	**McNemar's**	**p** = **0.001**		**p** = **0.008**	
**TC**	**Pass**	8	11	11	8
	**McNemar's**	**p** = **0.008**		**p** = **0.001**	
Standard plane		3D automatically-extracted			
**TV**	**3D operator-selected**	**Observer 1**		**Observer 2**	
		**Fail**	**Pass**	**Fail**	**Pass**
	**Fail**	5	6	3	5
	**Pass**	4	14	7	14
	**McNemar's**	**p** = **0.05**		**p** = **0.774**	
**TC**	**Fail**	5	3	9	2
	**Pass**	7	4	4	4
	**McNemar's**	**p** = **0.344**		**p** = **0.688**	
Standard plane		3D automatically-extracted			
**TV**	**2D manually-acquired**	**Observer 1**		**Observer 2**	
		**Fail**	**Pass**	**Fail**	**Pass**
	**Pass**	9	20	10	19
	**McNemar's**	**p** = **0.004**		**p** = **0.02**	
TC	**Pass**	12	7	13	6
	**McNemar's**	**p** = **0.000**		**p** = **0.000**	

## Discussion

This evaluation suggests that a 3D-DL algorithm can automatically extract standard planes from fetal head volumes of comparable quality to 3D-image planes selected by an operator from the same volume. No significant difference in image quality was demonstrated between 3D-modalities, although compared to corresponding 2D-planes, there was a significant reduction in quality.

This infers image quality limitations are related to acquired 3D-volumes used to train and test the algorithm. Quality of the 3D-volume and resultant planes may be limited by fetal head position within the maternal pelvis, and strong ossification of cranial bones causing shadowing artefact and decreasing visibility of intra-cranial structures.^19^ This is particularly relevant to TC-planes which had fewer adequate images in this study, likely due to the difficulty in visualising the cerebellum through obscuration of the posterior fossa. GA distribution of this dataset was wider than a typical antenatal ultrasound screening clinic because some participants were also required to have a paired fetal MRI scan as part of a wider research project,^20^ where fetal MRI is optimal at approximately 32-weeks GA. Yaqub et al., found their plane localisation was more accurate at the lower GA range of 23–27weeks,^21^ suggesting that such tools are better suited to earlier gestations where visibility of intra-cranial landmarks is optimised for screening. Image quality may also be limited using the X6-1 MHz matrix transducer which can be sensitive to fetal, maternal or operator movement during volume acquisition causing image degradation.^22^

Three-dimensional image quality may also be affected by volume acquisition technique, which may need refining for different GAs. Although an ideal method is yet to be agreed,^8^ with operator instruction, successful acquisition of 3D-volumes may be less dependent on the skill level of the operator than conventional 2D-imaging (e.g. providing the 3D-volume has covered the area of interest, the standard plane can be extracted).^19^^,^^23^ With the UK Sonographer workforce vacancy rate at 12.6% and ongoing recruitment challenges,^24^ automatic plane extraction tools show potential for improved clinical workflow by reducing scan acquisition time and standardising the technique. There is also wider potential globally in improving the accessibility of US for patients in areas where there may be a lack of expertise.

Acquiring 3D-volumes may be faster than current practice which requires the operator to navigate to the region of interest and obtain a specific 2D-plane.^22^^,^^25^, ^26^, ^27^ Benacerraf et al., reported the acquisition times for a routine 20-week fetal anomaly scan were halved using 3D-approaches, on average taking 6.6 min compared to 13.9 min for 2D.^28^ This could ease physical demands for US scanning.^29^ Advantages of 3D-fetal sonography in improved image quality/anomaly detection and acquisition time have been reported in published literature,^22^^,^^25^, ^26^, ^27^ although without further validation of 3D-neurosonography, it is only recommended that volumetric interpretation is used to compliment conventional 2D-assessment.^30^ Whilst outside of the scope for this study, future work may further develop the tool towards automatic extraction of additional planes (sagittal and coronal) required for complete fetal neurosonographic examination.^27^^,^^30^

High-quality fetal US images are essential to optimise visualisation, improve evaluation of anatomical structures and avoid litigation in cases of undetected anomalies.^31^ Image QA is subjective,^32^ and whilst attempts have been made to standardise evaluation, this is usually associated with biometrics.^13^^,^^33^ The USQA tool uses pre-defined criteria to help reduce subjectivity, and demonstrated moderate inter-observer agreement in assessment of overall image quality for 3D TV-planes. Image quality checklists may help guide observers undertaking image quality analysis; however, may not always reduce variation between observers' evaluations (e.g. weak inter-observer agreement for 3D TC-planes). This may be related to the small sample size, and/or the subjectivity of TC-plane assessment at later gestations (the TC-plane is infrequently imaged beyond 20-weeks unless a posterior fossa anomaly is suspected).^30^ Salomon et al., found that reviewers assessing fetal head image quality disagreed in over one third of cases, and the same reviewer could give varying judgements in up to 25% of cases,^13^ emphasising the subjectivity of review processes and the difficulties of proposing objective approaches. Further evaluation of the USQA software at earlier GAs using multiple reviewers is required to validate this approach to QA.

The accuracy of standard plane localisation using the ITN model was assessed quantitatively prior to image quality evaluation.^9^ A comparison of image quality scores with quantitative measurements was not conducted, however, even with a large rotation angle between the planes (δθ), the resultant images are often still visually similar (e.g. the image boundary region may be rotated significantly but the central brain region and key structures still remain intact and visually comparable). The algorithm may be more sensitive to alterations in the distance between the planes (δx) which could result in non-visualisation of key structures (thus failing to meet corresponding image quality criteria). These considerations highlight the differences between technical and clinical evaluation of DL-tools, and development in this area may help to further align the results.

## Conclusions

The clinical value of automatic-plane extraction tools for fetal head screening is not established. This study demonstrates that standard planes can be automatically-extracted from 3D-fetal head volumes to a similar quality of an operator-selected 3D-plane, however, quality of the 3D-volume from which the plane is extracted remains a limitation. Further work should focus on refining the algorithm using datasets from cohorts at lower gestations to improve this.

## Author's statement

All authors have made a significant contribution to the design, data collection and analysis and production of this manuscript.

## Data statement

The data sets collected and analysed during this study are available from the corresponding author on request.**:**

## Funding

This work was supported by the Wellcome Trust Council IEH Award [102431] for the Intelligent Fetal Imaging and Diagnosis project (www.ifindproject.com) and the Wellcome/EPSRC Centre for Medical Engineering [WT 203148/Z/16/Z]. The authors acknowledge support from the National Institute for Health Research (NIHR) Biomedical Research Centre at Guy's and St Thomas' NHS Foundation Trust and King's College London. The views expressed are those of the authors and not necessarily those of the NHS, the NIHR or the Department of Health. The study has been granted NHS R&D and ethics approval, NRES ref no = 14/LO/1086 and 07/H0707/105.

## Conflict of interest statement

None declared.
